# Burst-Suppression EEG Reactivity to Photic Stimulation—A Translational Biomarker in Hypoxic–Ischemic Brain Injury

**DOI:** 10.3390/biom14080953

**Published:** 2024-08-06

**Authors:** Alexandru-Cătălin Pâslaru, Alexandru Călin, Vlad-Petru Morozan, Mihai Stancu, Laurențiu Tofan, Anca Maria Panaitescu, Ana-Maria Zăgrean, Leon Zăgrean, Mihai Moldovan

**Affiliations:** 1 Division of Physiology—Neuroscience, Department of Functional Sciences, Carol Davila University of Medicine and Pharmacy, 050474 Bucharest, Romania; catalin.paslaru@umfcd.ro (A.-C.P.); vlad-petru.morozan@drd.umfcd.ro (V.-P.M.); stancu@bio.lmu.de (M.S.); laurentiu.tofan@drd.umfcd.ro (L.T.); anca.panaitescu@umfcd.ro (A.M.P.); ana-maria.zagrean@umfcd.ro (A.-M.Z.); leon.zagrean@umfcd.ro (L.Z.); 2 Department of Clinical Neurophysiology, King’s College Hospital NHS Foundation Trust, London SE59RS, UK; alexandru.calin@nhs.net; 3 Division of Neurobiology, Ludwig-Maximilian University, 80539 Munich, Germany; 4 Clinical Hospital of Obstetrics and Gynaecology Filantropia, 011132 Bucharest, Romania; 5 Obstetrics and Gynaecology Department, Carol Davila University of Medicine and Pharmacy, 050474 Bucharest, Romania; 6 Department of Neuroscience, University of Copenhagen, 2200 Copenhagen, Denmark; 7 Department of Neurology, Rigshospitalet, 2600 Glostrup, Denmark; 8 Department of Clinical Neurophysiology, Rigshospitalet, 2100 Copenhagen, Denmark

**Keywords:** rat, global cerebral ischemia, perinatal asphyxia, coma, clinical neurophysiology

## Abstract

The reactivity of an electroencephalogram (EEG) to external stimuli is impaired in comatose patients showing burst-suppression (BS) patterns following hypoxic–ischemic brain injury (HIBI). We explored the reactivity of BS induced by isoflurane in rat models of HIBI and controls using intermittent photic stimulation (IPS) delivered to one eye. The relative time spent in suppression referred to as the suppression ratio (SR) was measured on the contralateral fronto-occipital cortical EEG channel. The BS reactivity (BSR) was defined as the decrease in the SR during IPS from the baseline before stimulation (SR_PRE_). We found that BSR increased with SR_PRE_. To standardize by anesthetic depth, we derived the BSR index (BSRi) as BSR divided by SR_PRE_. We found that the BSRi was decreased at 3 days after transient global cerebral ischemia in rats, which is a model of brain injury after cardiac arrest. The BSRi was also reduced 2 months after experimental perinatal asphyxia in rats, a model of birth asphyxia, which is a frequent neonatal complication in humans. Furthermore, Oxytocin attenuated BSRi impairment, consistent with a neuroprotective effect in this model. Our data suggest that the BSRi is a promising translational marker in HIBI which should be considered in future neuroprotection studies.

## 1. Introduction

A transient decrease in oxygen or blood flow to the brain can result in hypoxic–ischemic brain injury (HIBI), a major cause of long-term disability and death. HIBI can occur at birth, or later in life, following cardiac arrest and resuscitation. The therapeutic approaches to HIBI remain so far unsatisfactory. An increasing number of experimental studies are targeting neuroprotective strategies in HIBI [1]. Nevertheless, their translational value to the clinical practice has proven so far to be limited [2]. One of the main challenges remains the lack of translational biomarkers to assess the degree of diffuse brain injury that can be used as outcome measures in therapeutic trials and as predictors of clinical recovery [3].

Recording the electrical activity of the brain using electroencephalography (EEG) remains central in the clinical assessment of HIBI, providing insights into the extent of the brain injury as well as the long-term monitoring of global brain function recovery by continuous or serial observations [4,5]. Although the EEG recording is readily available at the bedside, generating clinically meaningful biomarkers is complicated by the large amount of data reduction required. Consistent with the clinical thinking that the patient’s reactivity to external stimuli decreases with the increasing severity of their brain injury, there is a growing interest in methods quantifying the changes in the baseline EEG in response to standardized external stimulation [6,7], generally referred to as the EEG reactivity to that stimulation [8,9,10].

In conditions of a marked decrease in connectivity across large-scale brain networks, such as during general anesthesia [11], intermittent ‘bursts’ of EEG activity emerge on a suppressed low-voltage background activity, referred to as the burst-suppression (BS) pattern [12]. With increasing depth of anesthesia, the burst patterns occur less and less frequently as the EEG progresses to continuous suppression referred to as the isoelectric line [13], a pattern that can be fully reversible [14]. The relative EEG time spent in suppression (SR) [15] emerged as a surrogate marker for the depth of anesthesia across different anesthetics [16], driving closed-loop anesthesia delivery systems in rodents [17,18].

BS patterns induced by deep general anesthesia in humans were found to be reactive to external stimulation, especially to intermittent photic stimulation (IPS) [15,19] and electrical stimulation [20]. In clinical EEG studies, we found that intermittent electrical stimulation decreases the SR, and we introduced the measure of BS reactivity (BSR) [21]. Furthermore, we found that BSR to photic stimulation was decreased in children with acquired brain injury [22].

This study explored a novel approach for the functional assessment of experimental HIBI by cortical EEG. We aimed to assess brain functioning in deep anesthetic coma where the EEG shows BS patterns. The depth of anesthesia was controlled by measuring the SR. We evaluated the change in the SR during IPS by using BSR measures similar to our previous clinical studies [21,22]. Investigations were carried out in two well-established experimental models in rats: transient global cerebral ischemia (GCI), a model of brain injury after cardiac arrest [23,24], and perinatal asphyxia (PA), a model of birth asphyxia, which is a frequent neonatal complication in humans [25,26]. Furthermore, we explored the BSR changes following exposure to the biomolecule Oxytocin which was found to be neuroprotective in this model [26].

## 2. Materials and Methods

### 2.1. Animals and Experimental Design

In vivo electrocorticographic (ECoG) recordings were performed on adult male Wistar rats (200–400 g). The rats were randomly assigned to 3 experimental paradigms. In experiment 1, the rats were used to characterize ECoG reactivity to IPS over a wide range of anesthetic depths, up to BS. In experiment 2, BSR was measured before and 3 days after a 5 min episode of GCI. In experiment 3, BSR was measured at 2 months after experimental PA following treatment with either intranasal Oxytocin or a vehicle solution.

All experimental procedures were carried out during general anesthesia induced by either chloral hydrate (CHL, Sigma-Aldrich, Taufkirchen, Germany) or isoflurane (ISO, Rompharm, Romania). For CHL anesthesia, we administered an initial dose of 0.4 g/kg body weight i.p., followed by 0.1 g/kg every hour for maintenance. ISO was administered at a 1.0 L/min flow with a concentration of up to 4% through a Compact Gas Anesthesia System (Ugo Basile S.R.L., Gemonio, Italy) equipped with a debit meter accessory (Carbamed Digiflow, Heitenried, Switzerland). Body temperature was maintained at 37 °C using a heating pad (DC Temperature Control, FHC Inc., Bowdoin, ME, USA).

The rats were kept under standard laboratory conditions, with water and food ad libitum. Upon completion of the experiments, the rats were sacrificed by cervical dislocation under general anesthesia.

### 2.2. EEG Setup

Cortical EEG recordings were carried out via custom epidural electrodes [27]. Briefly, we implanted 4 wire electrodes through stereotactically drilled holes: 2 occipital electrodes at −6 mm posterior and ±3.35 mm lateral to bregma, and 2 anterior electrodes at +5 mm anterior and ±2.35 mm lateral to bregma. The electrodes were joined to an implantable multipin connector which was thereafter secured with dental cement (Unifast general-purpose acrylic resin; GC Dental Products Corp., Toriimatsucho, Japan). This electrode configuration allowed for a simple montage with two bipolar EEG channels: one across the right hemisphere (ECoG right) and one over the left hemisphere (ECoG left). This electrode configuration enables the recording of both the spontaneous global brain activity and visually evoked potentials (VEPs) [24,27].

Experimental recordings were carried out at least 3 days after the implantation to allow for impedance stabilization. In vivo ECoG recordings under anesthesia were obtained using an MP150 BIOPAC System—EEG100C modules at a 1–35 Hz bandpass filter—with the AcqKnowledge 4.2 Software (BIOPAC Systems, Inc., Goleta, CA, USA). The ground was placed at the mastoid. An electrocardiogram (ECG) channel was recorded in parallel with the EEG recordings. The recordings were digitized at 1000 Hz and stored for offline processing.

### 2.3. Transient Global Cerebral Ischemia (GCI) Model

Adult rats were exposed to a single episode of transient GCI induced using a variation of the ‘four-vessel-occlusion’ model [23] under general anesthesia [24,27]. In the first step, the vertebral arteries were exposed and electro-coagulated (Elektrochirurgie T120, Aesculap AG, Tuttlingen, Germany) through bilateral neck incisions. The carotid arteries were exposed through the same incisions and were reversibly occluded using atraumatic vascular microclamps. GCI followed by reperfusion was performed under EEG monitoring to validate the ischemic procedure by electrocortical suppression. We selected a GCI duration of 5 min, known to trigger neuronal death in vulnerable brain regions such as the hippocampus [28,29] while allowing for full cortical EEG recovery [30].

### 2.4. Experimental Perinatal Asphyxia (PA) Model

Day 6 postnatal (P6) pups of both genders originating from multiple mothers were exposed to hypoxia (9% O_2_) and hypercapnia (20% CO_2_) for 90 min to mimic the persistent impairment of gas exchange seen in PA, as previously described [25,31].

The PA experimental model was used to study the neuroprotective effects of Oxytocin, as previously described [26]. Thirty minutes before PA exposure, a group of pups received Oxytocin (Sigma-Aldrich, Taufkirchen, Germany) administered intranasally (0.02 IU/g body weight, dissolved in 2 µL sterile Ringer solution), using a pipette on the rhinarium, around the nostrils, avoiding direct application into the nostrils [32]. Control pups received only intranasal administration of the vehicle solution (Ringer solution only). The experiments were carried out in surviving adult rats at 2 months after PA. We randomly selected an equal number of male rats from the sham control group (PA-Sham) and the Oxytocin-exposed pups (PA-Oxy) for the cortical EEG reactivity studies. Note that in the PA model, the recordings were only performed in male rats to ensure a gender-controlled comparison across models.

### 2.5. EEG Reactivity Recordings

Measurements of cortical EEG reactivity to IPS were carried out during deep anesthesia by ISO, which allows for a faster anesthetic control than CHL. We measured the SR [33] ranging from 0% in continuous ECoG to 100% in the isoelectric line. We targeted a sedation level up to a SR of 50%, i.e., suppression periods at least as frequent as the burst activity, which can be readily identified by visual inspection of the live ECoG traces. This was typically achieved and maintained using a 4% ISO concentration. In control experiments, the ISO concentration was decreased toward 0 in 0.5% steps, allowing approximately 10 min for each step.

**Figure 1:**
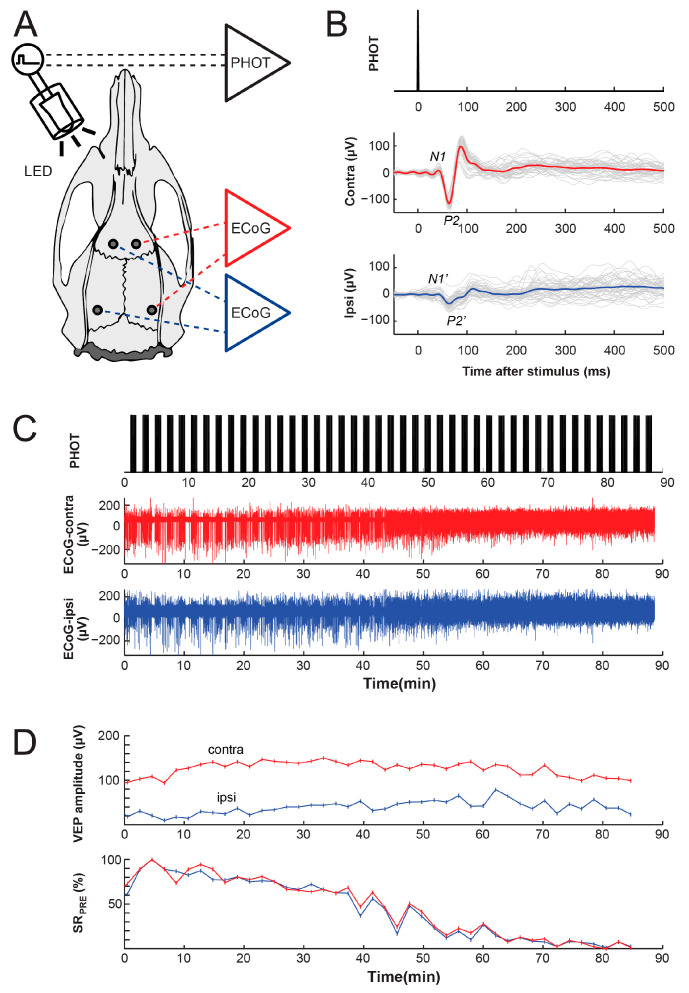
The experimental setup. (**A**) For cortical EEG recordings (ECoG), we implanted 4 epidural electrodes, 2 over the right hemisphere and 2 over the left hemisphere. Intermittent photic stimulation (IPS) was delivered near (<1 cm) the left eye while the other eye was held shut with opaque tape. The data are presented from a control experiment. (**B**) The visually evoked potentials (VEP) were obtained by averaging 30 photic (PHOT) stimuli within each stimulation trial (gray). The corresponding grand averages across the trials are depicted with thick lines for the contralateral and ipsilateral ECoG channels. The largest positive peak (downwards) was identified as P2, whereas the preceding negative peak (upwards) was labeled as N1. (**C**) We recorded multiple consecutive trials comprising of 60 s of IPS at 0.5 Hz alternating with 60 s stimulus-free periods. The recordings were carried out in two occipitofrontal channels, one over the brain hemisphere contralateral to the stimulation (contra) depicted in red, and one over the brain hemisphere ipsilateral to the stimulation (ipsi) depicted in blue. (**D**) The VEP amplitudes for each trial (top panel) were measured as the difference between N1 and P2 amplitudes on the contralateral channel, and between the corresponding N1′ and P2′ on the ipsilateral channel. The changes in the suppression ratio (SR) over the 30 s preceding each IPS trial (SR_PRE_) are depicted in the bottom panel over multiple consecutive IPS trials.

IPS was delivered near the left eye, while the right eye was held shut with opaque tape. Brief flashes (10 ms) were emitted at a frequency of 0.5 Hz via a white light-emitting diode (LED; 20 mcd maximum luminosity) using a custom-made stimulation device (Figure 1). Constant lighting conditions were ensured during the entire duration of the recordings. We implemented a stimulation protocol similar to our previous EEG reactivity studies in humans [21,22], with 60 s IPS epochs alternating with 60 s stimulus-free epochs. The timing of each LED pulse was recorded in parallel to the cortical EEG channel contralateral to the stimulus (ECoG contra) and the cortical EEG channel ipsilateral to the stimulus (ECoG-ipsi).

### 2.6. Cortical EEG Reactivity Measurements

**Figure 2:**
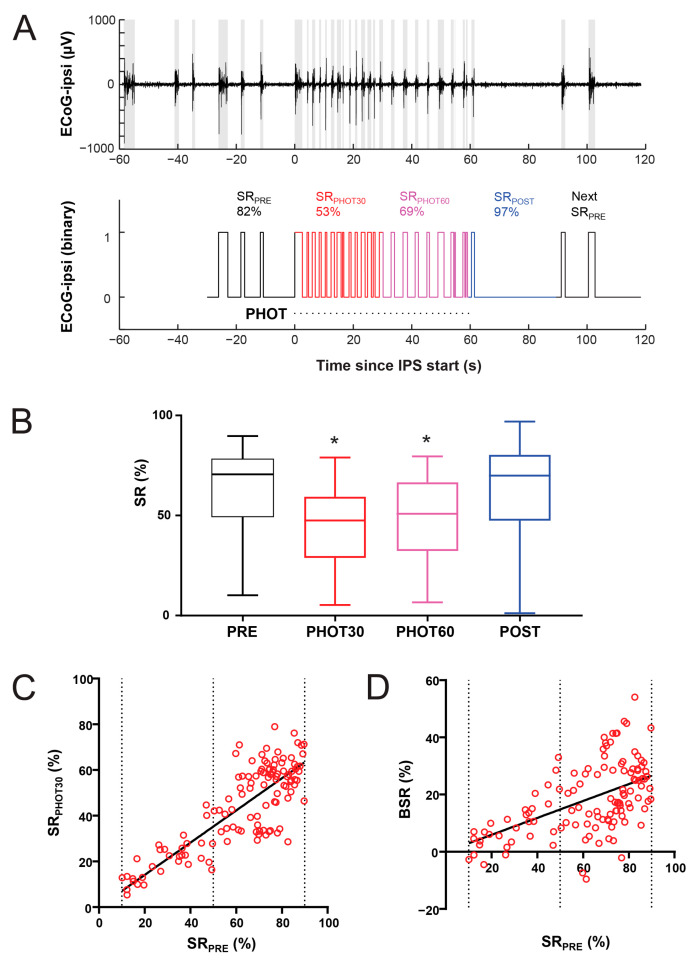
The burst-suppression reactivity (BSR) in controls. Measurements were carried out for each intermittent photic stimulation (IPS) trial on the ECoG channel ipsilateral to the photic stimulation (PHOT). (**A**) The relative time spent in suppression (SR) was measured in 4 consecutive 30 s epochs: PRE, the baseline preceding the onset of IPS; PHOT30, the first 30 s of stimulation; PHOT60, the last 30 s of stimulation; and POST, the 30 s following the stimulation. A further 30 s section with the next recorded PRE epoch is shown after POST. The top panel presents an ECoG recording example, where the burst activity was identified (intervals shaded in gray). The lower panel presents the same activity as a binary signal, where the periods of suppression are marked with 0 and the periods of bursts are marked with 1. The timing of each photic stimulus is marked underneath with a dotted line. (**B**) We included trials with an SR_PRE_ between 10% and 90% recorded from 10 rats. The resulting measurements (n = 120) are summarized as a box-and-whisker plot. The box extends from the 25th to 75th percentiles. The lines at the middle of each box are the median values. The whiskers go down to the smallest value and up to the largest. Asterisks indicate significant differences from SR_PRE_ (Wilcoxon *p* < 0.05). (**C**) The relationship between SR_PHOT30_ and SR_PRE_. The line depicts the equation SR_PHOT30_ = 0.7045 × SR_PRE_. (R^2^ = 0.6743). (**D**) The relationship between BSR and SR_PRE_, where BSR = SR_PRE_ − SR_PHOT30_. The line depicts the regression BSR = 0.2955 × SR_PRE_ (R^2^ = 0.3050).

The ECoG reactivity measurements were carried out offline for each trial comprising a 60 s stimulation epoch, preceded by at least 30 s of stimulus-free baseline and followed by at least 30 s of stimulus-free recovery (Figure 2).

EEG reactivity ultimately depends on the integrity of the peripheral pathway of the stimulated modality. Consequently, we first investigated the VEP by using triggered averaging of the ECoG on each channel. Each sweep, defined from 50 ms prior to the stimulus to 500 ms after the stimulus, was zero-centered prior to averaging. In control experiments (Figure 1), we found that 30 stimuli delivered during IPS were sufficient to allow for the identification of VEP under anesthesia. In accordance with previous studies using a similar montage [34,35], the large monophasic peak occurring 60–70 ms after the stimulus [36] was labeled as P2, whereas the preceding negative peak was labeled as N1. The VEP amplitude could therefore be calculated as the difference between the N1 amplitude and the P2 amplitude. We found that the VEP amplitude was larger on the ECoG channel contralateral to the stimulus than on the ECoG channel ipsilateral to the stimulus.

After assessing the VEP, the signals were downsampled to 200 Hz and refiltered using zero-phase filters to 1–35 Hz. The ECoG signals within each trial were binarized [17], distinguishing the periods of suppression (0) from the bursts (1). In contrast to the intracortical studies where the suppressions are ‘flat’ [37], the cortical EEG recordings over large interelectrode distances present a certain degree of ‘noise’, likely emerging from the asynchronous activity [38]. Our algorithm for adaptative thresholding of the BS signals was described in our previous studies in rats [30] and humans [21,22]. The minimal duration of suppression allowed was 0.25 s. The algorithm produced burst identification in good agreement with human observers.

The SR was calculated with a resolution of 30 s epochs, which was proven relevant for clinical reactivity studies [6]. Based on control experiments (see the Results Section for details), BSR was calculated as the decrease in the SR during the first 30 s of photic stimulation (SR_PHOT30_) from the SR prior to the stimulation (SR_PRE_). Thus, BSR was theoretically ranging from −100% to 100%. To avoid the potential confounding effect of the VEP occurrence on burst detection, the analysis was confined to the ECoG-ipsi.

### 2.7. Data and Statistics

Based on statistical power estimations from previous ECoG studies in rats under deep anesthesia, we considered sufficient a minimum of 5 rats for each experimental group [30,39]. ECoG recordings from a total of 25 rats were included in this study: 10 rats for the control experiments, 5 rats for the GCI experiments, and 10 rats for the PA experiments, of which 5 received sham treatment and 5 Oxytocin treatment. For each rat, we recorded multiple IPS trials, aiming to record at least 5 trials with an SR_PRE_ above 50%. The validity of all of the reactivity recordings was ensured by the presence of VEPs on the channel contralateral to the stimulus.

The stimulation triggering and data processing were carried out using custom-written MATLAB scripts (version 2024a for Windows, Mathworks Inc., Natick, MA, USA). The reactivity analysis was carried out per trial, each encompassing a 60 s IPS epoch. Considering that recordings were obtained during deep anesthesia and using implanted epidural electrodes, there were no confounding movement artefacts. Less than 2% of all trials were excluded for technical reasons.

The statistical analysis was performed using GraphPad Prism (version 10.2 for Windows, GraphPad Software Inc., La Jolla, CA, USA). Based on the findings from the control experiments, we introduced a standardized reactivity measure labeled as the burst-suppression reactivity index (BSRi), calculated as BSR / SR_PRE_ theoretically ranging from −1 to 1 (see the Results Section for details). As this study was focused on cross-sectional group differences, comparisons could be carried out by pooling all of the trials across the different rats per group. The results in numbers are given as the arithmetic mean ± standard error of mean. The number of trials included is mentioned for each group. Pairwise group comparisons were performed using nonparametric tests. All statistical tests used are specified.

All experimental procedures were carried out with the approval of the Local Committee for Animal Research Carol Davila University of Medicine and Pharmacy, Bucharest, Romania (7845/25.03.2021), and following the EU Directive 2010/63/EU on the protection of animals used for scientific purposes.

## 3. Results

### 3.1. Reactivity of Anesthetic Burst-Suppression Patterns to Photic Stimulation

To minimize the confounding effect of the VEP during IPS, we performed the analysis on the ipsilateral ECoG channel, which had the lowest VEP (Figure 1B). For each reactivity trial comprising 60 s of IPS (Figure 2A), the SR was measured relative to the onset of IPS in four epochs: the first 30 s before the onset of IPS (SR_PRE_), the first 30 s of IPS (SR_PHOT30_), the last 30 s of IPS (SR_PHOT60_), and the first 30 s after IPS (SR_POST_). Out of the control recordings carried out over a wide range of anesthetic depths (Figure 1D), 120 trials had an SR_PRE_ between 10% and 90% (Figure 2A). The mean SR_PRE_ was 62.66 ± 2.008%. The increasing bursting activity during IPS (Figure 2A) resulted in a decrease in the SR, as measured by an SR_PHOT30_ of 44.30 ± 1.674% (Wilcoxon *p* < 0.0001, Figure 2B). The decrease in the SR was sustained during the 60 s of stimulation, as measured by an SR_PHOT60_ of 48.84 ± 1.868% (Wilcoxon *p* < 0.0001, Figure 2B). The SR rapidly recovered following IPS, so that the SR_POST_ of 62.90 ± 2.117% was statistically undistinguishable from the SR_PRE_ (Wilcoxon *p* = 0.7666). This ensured both the stability of the anesthetic level during the trial, as well as the absence of large ‘after-effects’ of IPS.

The decrease in the SR during the first 30 s of IPS (SR_PHOT30_) was larger than during the subsequent 60 s of IPS (Wilcoxon *p* < 0.0001, Figure 2A,B). To measure the maximal effect of IPS on the SR, BSR was calculated as SR_PRE_- SR_PHOT30_. Across all of the investigated trials, the BSR was 18.37 ± 1.146%. The SR_PHOT30_ increased with the SR_PRE_ (Figure 2C) and the relationship could be reasonably described by linear regression (SR_PHOT30_ = 0.7045 × SR_PRE_, F = 2390, *p* < 0.0001, Figure 2C). Nevertheless, the BSR also increased with the SR_PRE_ (Figure 2D) and the relationship could also be reasonably described by linear regression (BSR = 0.2955 × SR_PRE_, F = 420.7, *p* < 0.0001, Figure 2D). These data suggest that the reactivity measurements increase with the deepening of anesthesia, as measured by the SR_PRE_.

### 3.2. Reactivity of Burst-Suppression Patterns Following Global Cerebral Ischemia (GCI)

**Figure 3:**
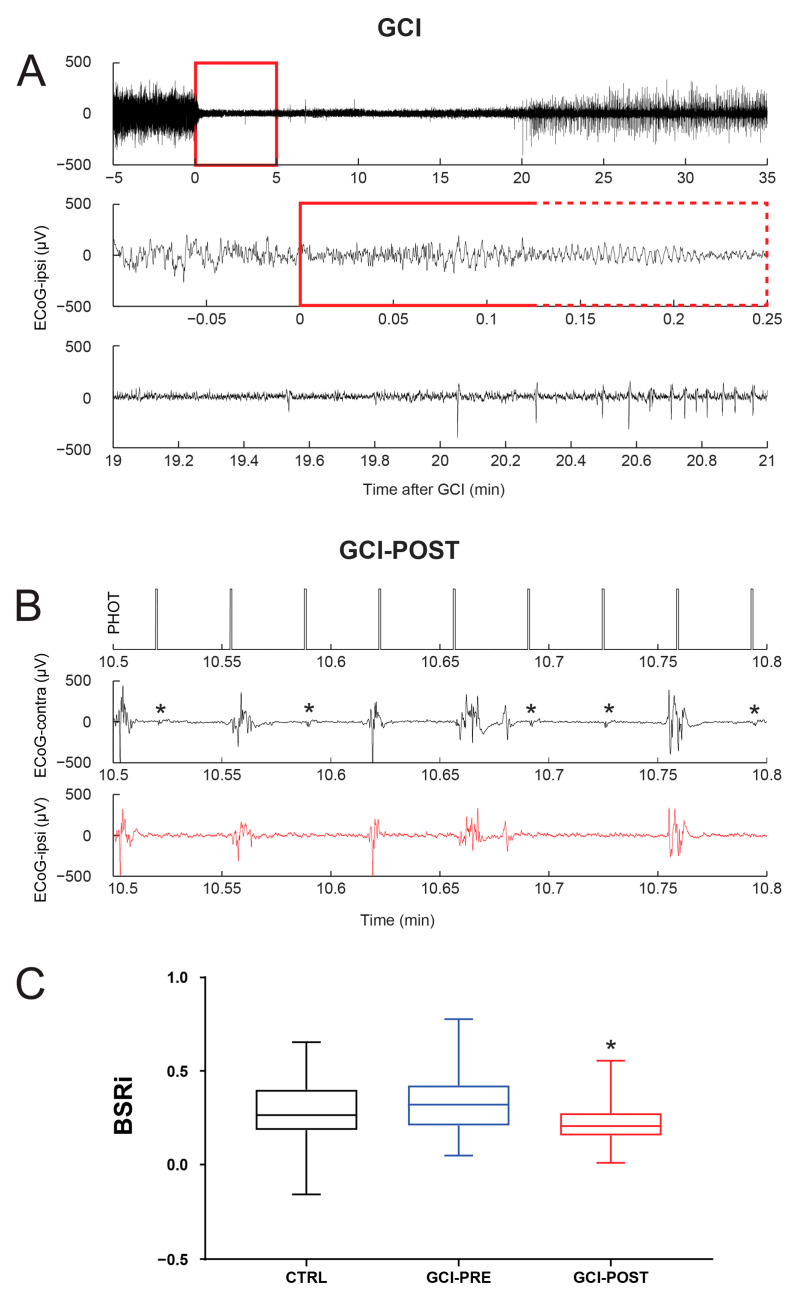
The reactivity of burst-suppression patterns following global cerebral ischemia (GCI). (**A**) ECoG recordings were carried out during a GCI protocol. After cauterization of both vertebral arteries, both carotid arteries were clamped (0 on X—time axis) and subsequently released after 5 min (top panel). The ischemia time is marked with a red box. Details are presented at the onset of ischemia (middle panel) and at the onset of ECoG recovery (lower panel). The red box in the middle panel marks the first 15 seconds of GCI, where the stippled part highlights the ECoG slowing before suppression (**B**) ECoG reactivity recordings were carried out 3 days after GCI at an SR_PRE_ > 50% induced by anesthesia. The recordings indicate the timing of the photic (PHOT) stimulus (top panel), ECoG on the channel contralateral to the photic stimulus (middle panel), and the ECoG channel ipsilateral to the stimulus (lower panel). Note that on the contralateral channel, the VEPs are readily identifiable (asterisk) even without averaging. (**C**) A comparison of the burst-suppression reactivity index (BSRi) between trials of the control (CTRL, n = 89, 10 rats), prior to GCI (GCI-PRE, n = 48, 5 rats), and after GCI (GCI-POST, n = 37, 5 rats) groups, for trials with an SR_PRE_ between 50% and 90%, presented as a box-and-whisker plot. The box extends from the 25th to 75th percentiles. The lines at the middle of each box are the median values. The whiskers go down to the smallest value and up to the largest. Significant differences from CTRL are indicated with asterisk (Mann–Whitney U, *p* < 0.05).

A group of five rats were subjected to a 5 min episode of GCI (Figure 3). Given the findings in the control experiments (Figure 2), to maximize the reactivity measurements, we adjusted the ISO concentration so that the SR_PRE_ was above 50% on the ECoG channel ipsilateral to the IPS, as appreciated visually at the time of recordings. Furthermore, we analyzed only trials with an SR_PRE_ between 50% and 90%. As indicated by our control experiments, BSR was expected to increase linearly with the SR_PRE_ (Figure 2D). To further account for the depth of anesthesia, we derived the standardized BSRi, calculated as BSR/SR_PRE_. Before GCI, the BSRi across 48 trials from the five rats was 0.3290 ± 0.02301 (Figure 3C). These measurements were similar (Mann–Whitney U, *p* = 0.2016) to the measurements from the 10 rats used in the control experiments (Figure 2) which had a corresponding BSRi of 0.2867 ± 0.01712, measured across 89 trials.

An example of 5 min GCI is presented in Figure 3A. Note that subsequent to electrocauterization of both vertebral arteries followed by clamping of both common carotid arteries, there was complete ECoG suppression within 20 s, consistent with our studies on the same model [24,30]. This was associated with ECoG slowing [39], as can be identified in Figure 3A’s middle panel (stippled box). The recovery of the full ECoG amplitude occurred within 30 min (Figure 3A, top panel) through a transient period of BS (Figure 3, lower panel), as previously characterized [30]. Following GCI, the rats resumed normal sleep–wake patterns, without signs of seizures.

At 3 days after GCI, the rats were re-anesthetized for reactivity measurements. Given the targeted depth of anesthesia to an SR_PRE_ > 50%, the VEP following flash stimulation was identifiable on the contralateral ECoG channel within the suppression periods, even without averaging (Figure 3B), consistent with our previous studies indicating that the flash VEP is resilient to GCI in this model [26]. We found that the ECoG remained reactive to IPS following GCI; however, the reactivity was impaired. The BSRi measured over 37 trials from five rats was 0.2272 ± 0.01919, which was lower than prior to GCI (Figure 3C, Mann–Whitney U, *p* = 0.0027).

### 3.3. Reactivity of Burst-Suppression Patterns Following Perinatal Asphyxia (PA)

**Figure 4:**
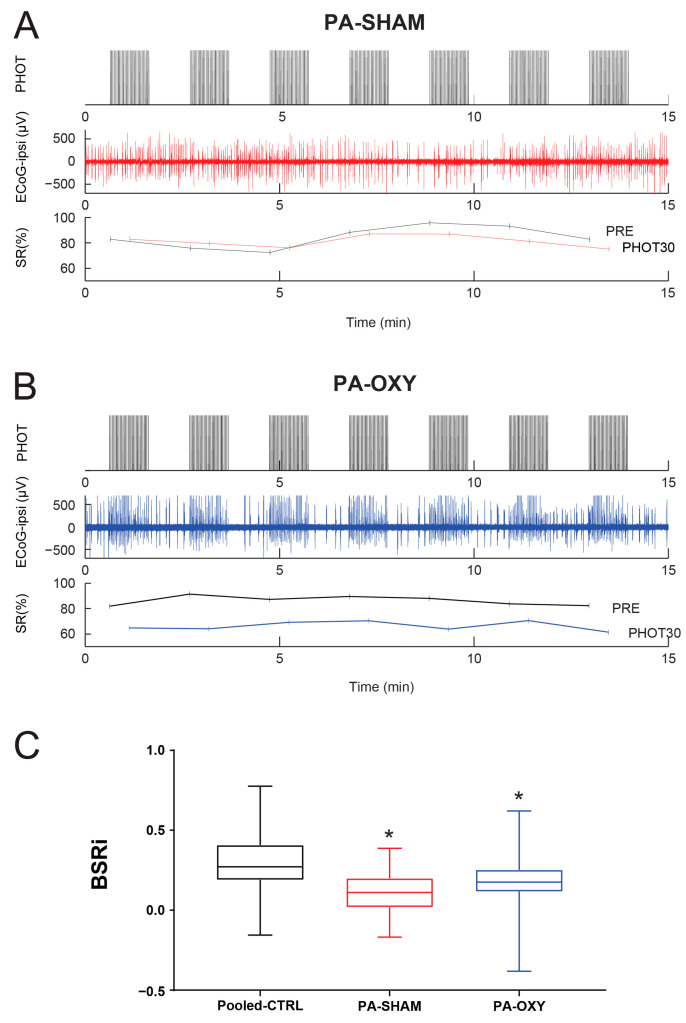
The reactivity of burst-suppression patterns following perinatal asphyxia (PA). A sequence of 7 trials of 1 min intermittent photic stimulation (IPS) alternating with 1 min stimulus-free epochs is presented in sham-treated rats (**A**) and Oxytocin-treated rats (**B**). The panels depict the timing of the photic (PHOT) stimulation (top), the ECoG ipsilateral to the stimulation (middle), and the suppression ratio (SR) calculated in the 30 s prior to IPS (PRE) and in the first 30 s of IPS (PHOT30). (**C**) A comparison of the burst-suppression reactivity index (BSRi) between trials with an SR_PRE_ ranging from 50% to 90% from 15 rats not subjected to PA (Pooled-CTRL, n = 137), 5 sham-treated rats following PA (PA-SHAM, n = 45), and 5 rats treated with Oxytocin (PA-OXY, n = 43), presented as a box-and-whisker plot. The box extends from the 25th to 75th percentiles. The lines at the middle of each box are the median values. The whiskers go down to the smallest and up to the largest values. Significant differences from Pooled-CTRL are indicated with an asterisk (Mann–Whitney U, *p* < 0.05).

Reactivity measurements were performed under anesthesia in rats at 2 months after PA. Consistent with our previous studies of the same model [26], all rats showed seizures during PA. We targeted a deep level of sedation at BS levels for the reactivity recordings, with an SR_PRE_ > 50%. We carried out consecutive trials of 1 min IPS, alternating with 1 min stimulus-free epochs (Figure 4). We aimed to record at least five trials within the targeted SR_PRE_ range, which typically resulted in a total recording time below 30 min. No seizures were identified in adult rats during the recordings, in neither sham-treated (Figure 4A) nor Oxytocin-treated (Figure 4B) rats. Furthermore, VEPs appeared well preserved on the channel contralateral to the stimulation, similar to the recordings following GCI (Figure 3B).

The measurements in PA adult rats were compared to all control measurements including the GCI controls (Figure 4C). The pooled control data across 15 adult rats, comprising 137 IPS trials with an SR_PRE_ between 50% and 90%, had a BSRi of 0.3015 ± 0.01379. Across 45 trials from sham-treated PA rats, the corresponding BSRi was 0.1070 ± 0.01905, which was considerably lower (Mann–Whitney U, *p* < 0.0001). Across 43 trials from five Oxytocin-treated rats, the BSRi was 0.1756 ± 0.02123, which remained lower than in the pooled controls (Mann–Whitney U, *p* < 0.0001).

Although the reactivity to IPS, as measured by the BSRi, was decreased following PA compared with controls, irrespective of treatment (Figure 4C), the reactivity was improved following Oxytocin in comparison to the sham treatment (Mann–Whitney U, *p* = 0.0063). The Oxytocin-triggered attenuation in reactivity impairment was clearly apparent at the deepest levels of sedation, where the difference in the SR between PRE and PHOT30 was larger following Oxytocin (Figure 4B) than after the sham treatment (Figure 4A).

## 4. Discussion

In this study, we propose a novel approach for assessing the impairment of brain function in rats subjected to experimental HIBI. We implemented a simple experimental setup to assess the cortical EEG reactivity to IPS in rats under deep anesthesia by analogy to our clinically available method [21,22]. The anesthetic depth was adjusted to achieve an SR of more than 50%, which is readily recognizable by visual inspection of the ECoG traces at the time of recording. To assess BSR to photic stimulation, it was necessary and sufficient to analyze only one ECoG channel, derived from two electrodes implanted occipitally and frontally over the brain hemisphere ipsilateral to the stimulation, to minimize the influence of VEPs [40]. The relatively large interelectrode distance ensured that global bursting activity was favored against local bursting activity [38,41].

Similar to the clinical findings, we found that BSR increased with the baseline SR just before the stimulation, referred to as SR_PRE_ [21,22]. Considering that SR_PRE_ is a marker for the depth of anesthesia in rodents [16], we derived an index of reactivity standardized by anesthetic depth, referred to as the BSRi, which facilitated the comparisons across a wide range of anesthetic concentrations and eliminated the need for strict anesthetic control. We aimed to record at least five trials of 60 s IPS at 0.5 Hz with an SR_PRE_ between 50% and 90%, which could be readily achieved within 30 min of recording, comparable to the conventional clinical EEG session [42]. Here, we chose IPS parameters similar to those in our clinical studies [21,22]. Nevertheless, for the assessment of reactivity in rodents, it was sufficient to focus only on the first 30 s of IPS. Intriguingly, 30 s epochs were also proven to be sufficient in other clinical reactivity methods [6]. This opens the possibility that the stimulation protocol should be further optimized to shorten the required recording time.

Impairment of cerebral energy metabolism during ischemia rapidly leads to neuronal depolarization, hyperexcitability, and ultimately, excitotoxic cell death [43]. We found that the BSRi was impaired in rats at 3 days following 5 min GCI [23] and at 2 months after PA [25,26]. These experimental models have well-characterized patterns of brain damage, ultimately accounting for the differential neuronal vulnerability to excitotoxic injury [44]. This injury mechanism in HIBI is considered epileptogenic, although less than in focal brain injury [43,45]. Monitoring the development of post-injury seizure activity requires long-term telemetric studies across different states of wakefulness [46], which were not performed in this study. Nevertheless, no seizures were observed that could have confounded the reactivity measurements in our study. This could be attributed to the fact that the investigations were carried out under deep sedation, which has a marked anti-seizure effect [47].

The investigations of brain reactivity depend on the integrity of the stimulated pathway. Somatosensory evoked potentials were found to be particularly sensitive to ischemic injury in humans [48], although they failed to predict long-term outcomes after perinatal hypoxia–ischemia in rats [49]. Here, we investigated the reactivity to photic stimulation. Although the analysis of the post-ischemic changes in the VEP was beyond the scope of this study, we found that the cortical VEP appeared largely preserved in the HIBI models we used (Figure 3B). The reduction in the SR during IPS primarily depends on its ability to trigger a widespread bursting activity over the cortex [50]. Consistent with previous observations in human subjects [22], we found corroborating evidence in rodent HIBI models that although the majority of bursts during IPS appeared to follow the cortical VEP, some VEPs failed to trigger bursts (Figure 3B). It was therefore likely that the impairment in the BSRi reflected an impairment in connectivity at the level of the large-scale cortical networks.

In patients with HIBI, the magnitude of the standardized BS reactivity to photic stimulation was found to depend on the severity of the injury measured on clinical scales [22]. In this study, we did not attempt to compare the BSRi with other biomarkers of injury [51]. Nevertheless, we found that in the PA model, the impairment in HIBI was slightly attenuated after the administration of Oxytocin, although it remained significantly reduced as compared to controls (Figure 4). Oxytocin is a neurohormone that has been found to be neuroprotective [52,53] in both in vitro models of transient hypoxia–ischemia [54,55] and in vivo PA models [26,56], as well as to have an anticonvulsant effect in an in vivo rat model [57]. As the BSRi biomarker primarily reflects the occurrence of bursting activity, the effect of Oxytocin may reflect an improvement in connectivity that could occur both at the synaptic level [37] and at the metabolic level [58]. Further studies should be performed to distinguish these mechanisms. Furthermore, it should be emphasized that here we focused on assessing the cortical EEG changes months after administration of Oxytocin in HIBI when the observed changes in the BSRi were not confounded by the presence of the exogenous Oxytocin, but rather reflected the neuroprotective EEG changes. It is thus reasonable to suspect that the BSRi could emerge as a general biomarker to assess the neuroprotective effects of biomolecules in HIBI [59].

Rodent cortical EEG activity shows oscillatory brain waves mostly in the 1–30 Hz frequency range, largely similar to recordings in humans [60], although a direct spectral analogy to the human frequency bands may not be entirely warranted [61]. Abnormal slowing of EEG (increased delta band activity) has long been associated with cerebral ischemia [62]. Focal delta activity rapidly recovers following intravenous administration of recombinant tissue plasminogen activator therapy in acute stroke [63], so it does not necessarily reflect neuronal damage. Previous studies from our group on the GCI model indicated that a similar slowing process can occur in rodents [39], as confirmed by observations in our current study (Figure 3A). This could be attributed, at least in part, to the synaptic connectivity impairment due to the release of adenosine [24,39], which is a retaliatory metabolite [64]. Furthermore, the connectivity impairment by adenosine was also found to promote BS activity during reperfusion [30]. In this study, we did not investigate the spectral changes within the bursting activity [39,65]. Nevertheless, as the assessments were carried out long after the HIBI episode, we do not expect that the adenosine-related changes played a decisive role in the observed reactivity impairment.

The translational value of the BSRi remains ultimately limited by the presence of BS patterns [66,67]. They can spontaneously emerge after HIBI [30,68], and their persistence is recognized as a marker of bad prognosis both after cardiac arrest [69,70] and in children with hypoxic–ischemic encephalopathy [71]. Nevertheless, the BS patterns are not detrimental per se as they can be completely reversibly induced during general anesthesia in both humans and experimental animals [72]. A recent clinical EEG method, referred to as the oscillatory macrostate analysis, was proposed to derive a binary signal from the spectral analysis of the EEG, from which a reactivity measure referred to as default EEG macrostate reactivity (DER) can be quantified similarly to BSR [73,74]. Further studies should be carried out to compare the translational value of these binary reactivity methods in rodent models.

## 5. Conclusions

We developed a rat model to assess the reactivity of burst-suppression cortical EEG patterns to photic stimulation. We derived the burst-suppression reactivity index (BSRi), a measure of reactivity standardized by anesthetic depth. We found that the BSRi was impaired in two established experimental models of HIBI. Furthermore, this impairment was attenuated in a well-characterized neuroprotective strategy. Our data suggest that the BSRi is a promising translational biomarker in experimental HIBI, which should be accounted for in future clinical trials exploring neuroprotection.

## 6. Patents

Part of the data analysis is protected by the European invention patent EP3646784B1 and the Romanian State Office for Inventions and Trademarks, patent OSIM Nr. 132025, RO 132,025 B1 to Termobit Prod SRL RO.

## Data Availability

The data that support the findings of this study can be made available from the authors upon reasonable request.
